# The Effects of Enhanced Enforcement at Mexico’s Southern Border: Evidence From Central American Deportees

**DOI:** 10.1007/s13524-020-00914-3

**Published:** 2020-09-10

**Authors:** Fernanda Martínez Flores

**Affiliations:** Migration and Integration Research Group, RWI – Leibniz-Institute for Economic Research, Hohenzollernstr. 1-3, 45128 Essen, Germany

**Keywords:** Immigration enforcement, Deportees, Central American migrants, Unauthorized, Transit countries

## Abstract

Immigration enforcement cooperation between final-destination and transit countries has increased in the last decades. I examine whether the Southern Border Plan, an immigration enforcement program implemented by the Mexican government in 2014, has curbed intentions of unauthorized migrants from El Salvador, Guatemala, and Honduras to migrate to the United States. I use the announcement of the Southern Border Plan to implement a difference-in-differences approach and compare the evolution of short-run intentions to engage in additional unauthorized crossings of Central American (treatment group) relative to Mexican deportees (comparison group). The findings suggest that increased enforcement in Mexico decreases the likelihood of attempting repeated unauthorized crossings.

**Electronic supplementary material:**

The online version of this article (10.1007/s13524-020-00914-3) contains supplementary material, which is available to authorized users.

## Introduction

**Fig. 1:**
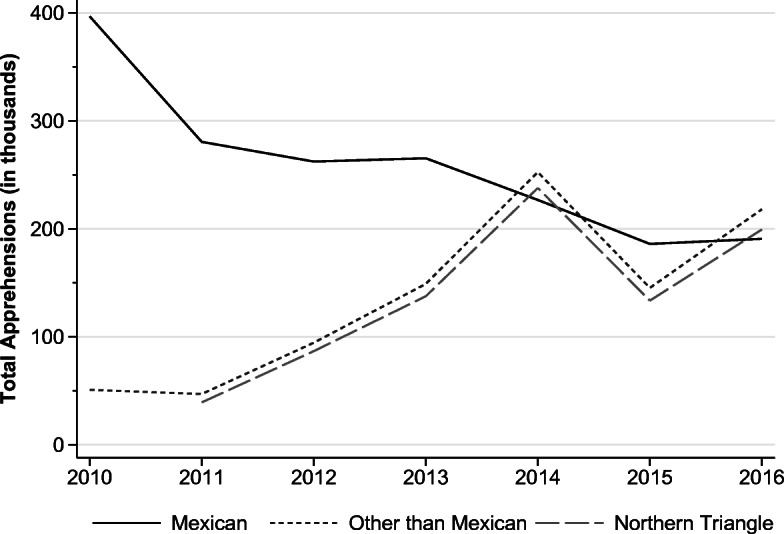
Total illegal alien apprehensions at the U.S. southwest border by fiscal year. *Source:* Author’s analysis based on data from U.S. Customs and Border Patrol (CBP) (2016).

About 50 million migrants worldwide lack the required legal permission to live and work in their chosen destination country (United Nations Office on Drugs and Crime (UNODC) 2010), implying that almost 20% of all international migrants are irregular (International Organization for Migration (IOM) 2017).^1^ Over the last decade, apprehension data along international borders have shown a growing number of asylum-seekers and economic migrants from developing countries arriving in developed countries, mainly in Europe and the United States.^2^ In Europe, irregular arrivals registered by land and sea tripled between 2013 and 2014 (100,000–280,000) and increased sixfold between 2014 and 2015 (1.8 million) (IOM 2016). In the United States, while the apprehension of Mexicans reached historical lows, the apprehension of Central American immigrants surged. Figure 1 illustrates that from 2010 to 2014, the apprehensions of non-Mexicans increased fivefold, mainly driven by citizens from Central America’s Northern Triangle: El Salvador, Honduras, and Guatemala.

Because of the increase in irregular migration, final-destination countries have partnered with transit countries to step up their immigration enforcement measures. Cooperation between destination and transit countries has taken the form of repatriation agreements, enhancing border controls, training personnel, and providing technical and financial assistance (Djajić and Michael 2014). Some examples of bilateral cooperation are the Treaty on Friendship, Partnership and Cooperation signed by Italy and Libya in 2008, and the EU-Turkey Statement signed in 2016, both aiming at increasing enforcement while migrants are in transit.^3^

A large body of literature has analyzed whether immigration enforcement in the destination country impacts unauthorized migration—mostly of Mexican workers to the United States^4^—but there is no empirical evidence on the effect of cooperation between destination and transit countries to curtail unauthorized immigration. To the best of my knowledge, this study is the first to analyze empirically whether tougher enforcement measures in transit countries curb migration intentions of unauthorized migrants.^5^

Theoretically, Djajić and Michael (2014) showed that destination countries can control unauthorized immigration by providing financial aid to transit countries to step up their enforcement actions. Djajić (2017) indicated that the additional disutility faced by migrants in transit countries has the potential to deter unauthorized migration. More controls in transit countries influence the destination country’s immigration policy by increasing the effectiveness of border controls and discouraging individuals to migrate in the first place. Moreover, the empirical evidence on bilateral migration flows suggests that immigration policies set by other countries have important cross-country spillovers (see, e.g., Bertoli and Moraga 2015; Boeri and Brücker 2005; Giordani and Ruta 2013). Against this background, I evaluate whether the Southern Border Plan (SBP), an immigration enforcement program announced by the Mexican government in 2014 aimed at decreasing transit migration flows to the United States, has had an impact on remigration intentions of Central American deportees.

The existing empirical literature presents mixed evidence of the effect of immigration enforcement on unauthorized migration.^6^ Some studies focusing on enforcement in the United States used changes in Border Patrol (BP) watch hours and aggregate apprehension data but found no or little evidence that increased enforcement deters Mexican undocumented migrants (see, e.g., Angelucci 2012; Dávila et al. 2002). Similar results were found by studies focusing on national policies, such as the Immigration Reform and control Act implemented in 1986, which made it illegal to employ unauthorized migrants (see, e.g., Cornelius 2001; Donato et al. 1992; Kossoudji 1992), and Operation Streamline implemented in 2005, which criminalized crossing the border without the corresponding documentation (see, e.g., Amuedo-Dorantes and Pozo 2014; Cañas et al. 2011). These studies found evidence of other unintended consequences, such as mounting migration costs, higher risks of injury and death (Gathmann 2008; Massey et al. 2002), a rise in smuggler markets (Kossoudji 1992), and a shift from temporal to permanent migration patterns (Angelucci 2012; Massey and Pren 2012; Massey et al. 2016).

In contrast, studies focusing on state-level policies have found that enforcement curtails irregular migration. Examples include the Legal Arizona Workers Act announced in 2007, which required all employers in Arizona to check for employment eligibility; and the *Arizona SB 1070* announced in 2010, which allowed the local police to verify the immigration status of an individual during a lawful stop. State policies are effective in (1) deterring unauthorized migrants in the short run (Amuedo-Dorantes et al. 2013), (2) decreasing the proportion of noncitizen Hispanic population in the state (Bohn et al. 2014; Lofstrom et al. 2011), and (3) changing immigration and locational choices of new Mexican immigrants (Hoekstra and Orozco-Aleman 2017).

Although empirical studies provide evidence on the effect of increased enforcement in destination countries, the results cannot be generalized to transit countries. Understanding how enforcement in transit countries curbs migration is a key aspect for migration policies aimed at reducing the flow of unauthorized migrants. I contribute to filling this gap by evaluating whether a more restrictive immigration policy in Mexico has the potential to stem the tide of irregular immigrants in transit to the United States.

Specifically, using exogenous variation provided by the introduction of the SBP, I test whether increased immigration enforcement in Mexico has an impact on the likelihood of reporting intentions to remigrate of Central American migrants apprehended while being in transit. I combine surveys on Central American and Mexican irregular migrants apprehended and deported by Mexican and U.S. authorities from 2012 to 2016. Using a difference-in-differences (DiD) approach, I compare the evolution of short-run intentions to engage in additional unauthorized crossings of Central American relative to Mexican migrants.

Two concerns are worth clarifying: (1) data on deportees may not be representative of the entire unauthorized population, and (2) migration intentions may not translate into future crossings. Ideally, this analysis would be conducted using data on observed migration outcomes of all Central Americans who could potentially engage in unauthorized crossings. Yet, no survey data available allow for such an exercise. Using data on deportees allows evaluating whether additional enforcement affects recidivism intentions of individuals who have been exposed to these measures and who have shown a propensity to migrate (see, e.g., Amuedo-Dorantes and Pozo 2014). Even if the results cannot be generalized to the population of Central American undocumented immigrants, they are informative about how unauthorized migrants respond to enforcement measures in Mexico.

With respect to the second concern, studies show that migration intentions are highly correlated with migration outcomes and are a good predictor of future emigration (Creighton 2013; Docquier et al. 2014; van Dalen and Henkens 2013). There is no reason to suspect that individuals misstate their intents because the surveys are collected in the origin country and not conducted by authorities related to the apprehension-deportation process.

Additionally, if enforcement is effective, it should start by impacting migration intentions (Amuedo-Dorantes and Pozo 2014). In fact, according to Bailey et al. (2016), although the likelihood of apprehension at the U.S. border increased from 40% to 55% from 2010 to 2015, the deterrence rate rose from 10% to 60% in the same period. Thus, the sharp drop in apprehensions of Mexicans at the U.S. border is driven not only by an increase in the apprehension probability but also by an increase in the number of people being deterred from trying to cross the border (Alden 2017). Therefore, even if I cannot overcome the limitations associated with using migration intentions instead of actual migration, the decrease in intentions is likely to be associated with a decrease in attempts to cross the border, even if this is not a one-for-one relationship.

External validity to other countries and migration routes remains an open question, yet my findings suggest that enforcement of bilateral cooperation between Mexico and the United States has the potential to deter unauthorized immigrants in the short run. The results show that the SBP significantly decreases the likelihood of recidivism. Migrants who have access to a network in the United States are less deterred by the SBP. Furthermore, migrants who were not apprehended in Mexico but in the United States do not respond to the program. Finally, the results show that total apprehensions of Central Americans relative to Mexicans at the U.S. border decreased after the SBP was implemented.

## The Southern Border Plan

Thousands of Central American immigrants, mainly from the Northern Triangle, transit through Mexico and travel north to the United States every year.^7^ Spanning 209 km, the Mexican southern border with Guatemala and Belize has 11 formal border controls for terrestrial crossings, but authorities estimate the existence of more than 700 points of informal crossings (Secretaría de Gobernación (SEGOB-CAIMFS) 2015). Because of its porosity, about 95% of migrants in transit cross the border without the proper legal documentation (SEGOB 2014b). Under the Central America-4 (CA-4) visa system, Guatemala, Honduras, El Salvador, and Nicaragua do not require each other’s citizens to present visas or passports but only their identity cards. As a result, both arriving at and crossing the Guatemala-Mexico border is simple. Once in Mexico, migrants make their way toward the U.S. border along established transit routes or atop freight trains known as *La Bestia* (Dominguez Villegas 2014).

The porosity of Mexico’s southern border led to increased cooperation in border security between Mexico and the United States since 2007 when the Mérida Initiative^8^ was launched. Under this initiative, the Mexican government committed to increasing its efforts to improve security at the southern border. Since 2013, the U.S. State Department has provided Mexico with $24 million (in U.S. dollars) in equipment and training assistance, which includes mobile kiosks, canine teams, and training for officials of the National Migration Institute (INM; *Instituto Nacional de Migración*); and has targeted more than $75 million USD to improve security at Mexico’s southern border (Seelke and Finklea 2017).

Despite the investments, the flows of Central American migrants increased. From 2008 to 2011, the estimated annual flow of Central American migrants was about 135,000.^9^ Economic crises, increased levels of poverty, inequality, and different forms of violence in the Northern Triangle resulted in an unprecedented flow of women and unaccompanied children arriving at the U.S.-Mexico border in 2014. During this year, the estimated flow of migrants tripled, reaching a total of 392,000.

As a response, both the United States and Mexico increased border security to detain and remove unauthorized immigrants. For Mexico, the cooperation on enforcement implied increasing its removal efforts, disrupting traditional and well-developed migrant routes, and installing new checkpoints to apprehend and deport unauthorized immigrants. On July 7, 2014, former Mexican president Enrique Peña Nieto announced the introduction of the Southern Border Plan (*Plan Frontera Sur*),^10^ a formal strategy to stem the flows of undocumented Central American immigrants in transit through Mexico.

Despite the stated objectives of the SBP (see SEGOB 2014a), the Mexican government focused on the apprehension and repatriation of undocumented migrants (Castañeda 2014). Mexico has chosen to focus its enforcement actions in the country’s interior by implementing security belts and increasing the number of raids and mobile checkpoints in strategic places (SEGOB 2014a). Immigration enforcement in Mexico is, to a certain extent, similar to U.S. enforcement at the state level and operates on a “show your papers” mode.^11^

**Fig. 2:**
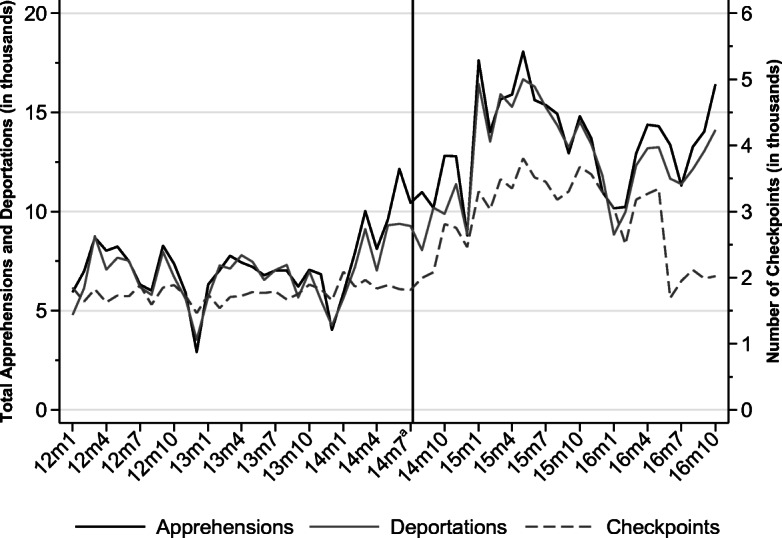
Deportations, apprehensions, and checkpoints by Mexican authorities. *Source:* Author’s analysis using data requested from the INM. ^a^ Month when the SBP was introduced.

Figure 2 shows monthly apprehensions and deportations by Mexican authorities from 2012–2016. Mexico apprehended 119,714 Central American migrants in 2014, the vast majority of whom were citizens of the Northern Triangle, which represents a 48.2% increase from 2013, when authorities apprehended only 80,757 migrants. The number of deportations in Mexico is almost perfectly correlated with the number of apprehensions (ρ = *.*98), showing that Mexico apprehends migrants with a catch-and-release policy. The figure further shows that apprehensions increased shortly before the SBP was announced, which can be explained by an increasing inflow of Central American immigrants in transit and not necessarily by increased enforcement in Mexico before the SBP. To support this argument, Fig. 2 also plots the monthly number of immigration checkpoints operated by the INM. The number of checkpoints increased immediately after the announcement of the SBP implementation in July 2014 and reached the highest number during 2015. On average, before the start of the SBP, 1,770 checkpoints were installed every month. After the initiation of the SBP, the monthly average increased by almost 50% (2,630 checkpoints).

Figure A1 in the online appendix shows that before 2014, while the U.S. BP apprehended on average 13,000 migrants every month, apprehensions in Mexico were about half the size. Right after the implementation of the SBP, U.S. BP apprehensions dropped, and apprehensions by Mexican authorities increased. In 2015, apprehensions in Mexico even surpassed U.S. apprehensions by 12%. In 2016, the number of checkpoints installed in Mexico decreased, and Fig. 1 shows that apprehensions by U.S. authorities increased. This implies that the ability of the United States to curb undocumented migration inflows partially depends on the extent to which Mexico controls transit flows originating from other Latin American countries.

## Identification Strategy

Using a DiD approach, I compare remigration intentions of migrants from the Northern Triangle in transit through Mexico to the United States, apprehended and deported by Mexican authorities (treatment group), with remigration intentions of Mexicans apprehended and deported by U.S. authorities (control group). I exploit that Mexican and Central American immigrants in the United States share similar characteristics, such as destination choice, age, gender, labor force participation, and occupation, to define the treatment and control groups (see, e.g., Brick et al. 2011).

To identify the causal effect of increased immigration enforcement in Mexico, I use the SBP as a source of exogenous variation. Whereas changes in immigration enforcement in the United States would affect both groups equally, unauthorized Central American migrants face tougher enforcement measures after July 2014 in Mexico. Mexicans, in contrast, are not affected by the new enforcement measures proposed by the SBP. The DiD strategy is implemented by estimating:

1 $$ {Y}_{idts}=\upalpha +\upbeta \left(C{A}_i\times PostSB{P}_t\right)+{\uptheta}^{\prime }{\mathbf{X}}_i+{\upkappa}_s+{\upgamma}_d+{\uplambda}_t+{\updelta}_d\mathbf{t}+{\upkappa}_s\mathbf{t}+{\upvarepsilon}_{idts}. $$

The outcome of interest *Y*_*idts*_ is a binary variable that takes the value of 1 if the individual *i* who migrated from department^12^*d*, in quarter *t*, and was apprehended in state *s*, reported that s*/*he will try another crossing in the future; and 0 otherwise. I differentiate between *short-run intentions*, which indicate that the migrant intends to return to the United States in less than three months, and *long-run intentions*, which indicate whether the migrant intends to return sometime.

*CA* is a binary variable that equals 1 if the individual is Central American (treatment group) and 0 if the individual is Mexican (control group). The dummy variable *PostSBP* takes the value of 1 if the individual started his/her trip after the SBP was announced (third quarter of 2014). The coefficient of interest, β, reflects the change in reported intentions for the treatment group relative to the control group after the program was announced.

The vector **X** includes the following variables: first, individual demographic characteristics likely to affect the decision to migrate, such as age, gender, English skills, education level, marital status, whether the migrant is the household head, and household size; second, characteristics related to the migration event itself, such as total money spent, borrowing money for the trip, using a smuggler, and the number of persons traveling together; third, an indicator of having access to a network in the United States measured by the presence of family or friends in the United States; last, control variables for previous migration experience, such as the number of previous crossings and deportations.

I include dummy variables for department of origin, γ_*d*_, to capture time-invariant characteristics that may relate to migration patterns, such as regional historical emigration levels. Apprehension state fixed effects, κ_*s*_, capture time-invariant characteristics related to an environment hostile to transit migration. Quarter fixed effects, λ_*t*_, control for shocks that affect the desire to migrate to the United States, such as changes in immigration enforcement in the United States.

To account for different pre-treatment trends for treated and control individuals, I include time trends, δ_*d*_**t**, for each origin department. This interaction captures changing socioeconomic and political characteristics correlated with the decision to migrate, such as escalating crime and/or unemployment rates. I further control for apprehension state-level time trends, κ_*s*_**t**, to capture changing conditions in the state where the migrant was apprehended, such as increasing immigration enforcement measures at the state level or increasing hostility against migrants. The standard errors are clustered at the origin-department level to account for correlation within the geographical units where migrants originated (Cameron and Miller 2015).

Several concerns arise in this framework. The main identifying assumption is that in the absence of the SBP, remigration intentions of the treatment and control group would have followed parallel trends. Without additional enforcement measures in Mexico, the evolution of reported intent of recidivism should not systematically differ among Central American and Mexican deportees. To show that both groups follow similar pre-treatment trends, I provide descriptive evidence on the evolution of remigration intentions. I later estimate the model using a placebo treatment.

Two main arguments could call into question the validity of the control group. On the one hand, Mexican unauthorized migrants may adjust their intentions to remigrate if fewer Central Americans arrive at the U.S. border after implementation of the SBP. On the other hand, Central American migration has been increasingly driven by humanitarian reasons.^13^ A growing number of Central American minors and families do not seek to avoid authorities at the U.S. border; instead, they surrender to the first U.S. authority or request asylum at U.S. entry points (U.S. Department of Homeland Security (DHS) 2017). Although the descriptive evidence shows that Mexicans do not alter their intentions to remigrate and Eq. (1) controls for worsening conditions in the sending regions, I also conduct the analysis using an alternative control group to address these concerns.

Stricter immigration enforcement in Mexico induces additional selection in the deportees’ sample in terms of observable and unobservable characteristics. Observed characteristics of Central American deportees are very similar before and after the start of the SBP in Mexico and the United States (see Tables A1 and A3 in the online appendix). Changes in selection on unobservable characteristics cannot be ruled out. However, if increased enforcement measures cause more motivated or risk-loving individuals to migrate, the estimated effect of the SBP on remigration intentions would represent a lower bound. To show that the estimated results are not purely driven by changes in the sample composition, I conduct an exercise using aggregate data on apprehensions of Central American undocumented migrants by Mexican and U.S. authorities.

## Data and Descriptive Statistics

I combine data from the Survey on Migration at Mexico’s Southern Border (EMIF South) and the Survey on Migration at Mexico’s Northern Border (EMIF North). The EMIF data are cross-sectional surveys conducted by *El Colegio de la Frontera Norte* (COLEF) and supported by multiple Mexican governmental organizations.^14^ The surveys are conducted along Mexico’s northern and southern borders as well as in specific locations where deportees are returned. The EMIF South focuses on migration flows from the Northern Triangle to Mexico or the United States. The EMIF North focuses on migration flows of Mexicans to the United States.^15^

I focus on a subset of the EMIF surveys spanning 2012–2016: Central Americans (EMIF South) deported by Mexican authorities and Mexicans (EMIF North) deported by U.S. immigration authorities. In addition, I use data on Central Americans deported by U.S. authorities to test whether the estimated effects also hold for migrants who were not apprehended in Mexico. The EMIF South and North are not designed identically, but most questions are comparable. Deportees are surveyed after repatriation at the first point of arrival and report information on the trip, apprehension and deportation process, and future migration plans.

The main advantages of using the EMIF data on deportees are twofold. First, the data allow the determination of whether an individual has the corresponding papers to travel and work both in the United States and Mexico—that is, whether the individual is unauthorized.^16^ Second, the data allow the identification of the exact date when the migrants started their trip. Thus, individuals who started their trip before and initiation of the SBP can be differentiated from those who started post-SBP. One of the main shortcomings of the EMIF data is that the reported weights are estimates of the flows passing through sampling zones; the true weights remain unknown because the underlying population is unknown (National Research Council 2013).

To consolidate a group of migrants who share similar characteristics, I first restrict the sample to individuals who started their trip to the United States sometime during the period January 2012 to December 2016 and reported residing in their country of birth (with 217 Central American and 4,789 Mexicans who reported the United States as their place of residence being dropped from the sample).^17^ Second, I drop from the sample Central American individuals who crossed the Mexico-Guatemala border via air or sea because they are less likely to cross the border without documents (49 observations). Third, I exclude Mexican migrants who entered the United States legally (644 observations) or crossed the border via air (1,335 observations). The final sample consists of 32,041 observations, of which 70% are Central American and 30% Mexican. The data are weighted using an expansion factor to account for differential sampling probabilities. The Central American sample consists of 28% Salvadoran, 30% Guatemalan, and 42% Honduran deportees.^18^

Table 1 presents the pre-program descriptive statistics for Central American (treatment) and Mexican deportees (control).^19^ Despite the statistically significant differences in individual characteristics between Central American and Mexican deportees, these are negligible in most cases. The table shows that 87% of deportees are male, and the mean age is 29. Deportees come from households with five members, were employed in their country of origin before migration (57%), and reported having family or friends living in the United States (63%). Some differences between Central American and Mexican deportees are observed. Central Americans are less likely to be married (41% vs. 57%) and to be the household head (41% vs. 58%). The majority of Central Americans have completed only primary education or less (64%), and the majority of Mexicans have completed low-secondary education (49%).

**Table 1:** Pre-program descriptive statistics

	All Deportees		Treatment		Control		
	Mean	SD	Mean	SD	Mean	SD	∆ Mean
Dependent Variables							
Intent to remigrate (short run)	0*.*405	0*.*491	0*.*537	0*.*499	0*.*362	0*.*481	–0.175***
Intent to ever remigrate	0*.*650	0*.*477	0*.*756	0*.*430	0*.*615	0*.*487	*−*0*.*141***
Independent Variables							
Male	0*.*868	0*.*338	0*.*862	0*.*345	0*.*870	0*.*336	0*.*008
Age	28*.*721	8*.*071	27*.*104	7*.*172	29*.*253	8*.*277	2*.*149***
Speaks English	0*.*104	0*.*305	0*.*006	0*.*079	0*.*136	0*.*343	0*.*130***
Education							
Primary education or less	0*.*386	0*.*487	0*.*637	0*.*481	0*.*303	0*.*460	*−*0*.*334***
Secondary education	0.423	0.494	0.210	0.407	0.493	0.500	0.283***
High school	0*.*171	0*.*377	0*.*143	0*.*350	0*.*181	0*.*385	0*.*038***
Tertiary education	0*.*020	0*.*140	0*.*010	0*.*101	0*.*023	0*.*151	0*.*013***
Married	0*.*532	0*.*499	0*.*410	0*.*492	0*.*572	0*.*495	0*.*162***
Head	0*.*539	0*.*498	0*.*409	0*.*492	0*.*582	0*.*493	0*.*173***
Household size	5*.*003	2*.*168	5*.*571	2*.*108	4*.*816	2*.*155	–0*.*754***
Employed before migration	0*.*569	0*.*495	0*.*679	0*.*467	0*.*533	0*.*499	–0*.*146***
Has family/friends in the United States	0*.*629	0*.*483	0*.*608	0*.*488	0*.*635	0*.*481	0*.*027*
Money spent (in $1,000 USD)^a^	1*.*618	1*.*766	0*.*560	1*.*031	1*.*967	1*.*818	1*.*407***
Borrowed money to cross	0*.*643	0*.*479	0*.*369	0*.*482	0*.*733	0*.*442	0*.*364***
Used a coyote	0*.*485	0*.*500	0*.*179	0*.*383	0*.*586	0*.*493	0*.*408***
People traveling together	2*.*971	5*.*089	1*.*202	2*.*005	3*.*553	5*.*633	2*.*350***
Traveled with children	0*.*041	0*.*198	0*.*030	0*.*171	0*.*045	0*.*206	0*.*014***
Previous number of crossings	0*.*809	2*.*004	0*.*152	0*.*437	1*.*026	2*.*256	0*.*874***
Country of origin							
El Salvador	0*.*064	0*.*244	0*.*257	0*.*437	––	––	––
Guatemala	0*.*076	0*.*264	0*.*305	0*.*460	––	––	––
Honduras	0*.*108	0*.*311	0*.*438	0*.*496	––	––	––
Mexico	0*.*752	0*.*432	––	––	1*.*000	0*.*000	––
Number of Observations	18,462		12,172		6,290		

*Notes:* Pre-program is defined as the interval between January 1, 2012 and June 30, 2014. The treatment group is defined as individuals deported by Mexican authorities to their origin countries: El Salvador, Honduras, and Guatemala. The control group defined as Mexicans deported by U.S. authorities. The last column shows the difference in mean values between Central American and Mexican deportees.

^a^Calculated using predicted travel costs plus the reported smuggler fees.

*p < .05; ***p < .001

Not surprisingly, both groups differ with respect to the trip characteristics. Compared with Mexicans, Central Americans travel in smaller groups and are less likely to borrow money for the trip and to use a smuggler. The amount of money spent on the trip is calculated using a prediction of total travel expenditures plus the reported expenditures on smuggler’s fees. Central Americans spend an average of $560 USD, compared with $1,967 USD for Mexicans. Expenditures for Central American deportees are much lower because they were apprehended at an early stage of their trip. Central Americans apprehended in the United States reported similar trip characteristics as Mexicans in terms of smuggler use, group size, and borrowing money. They also reported higher expenditures than Mexicans, at about $3,300 USD (see Tables A2 and A3 in the online appendix).

Table 1 further shows that among Central Americans, 54% reported intending to cross the border again in the short run, whereas 76% reported that they will attempt to cross the border in the long run. For Mexicans, these shares are 36% and 62%, respectively. The difference in levels for both groups is not a problem for identification if both groups follow a similar trend in the pre-program period.

**Fig. 3:**
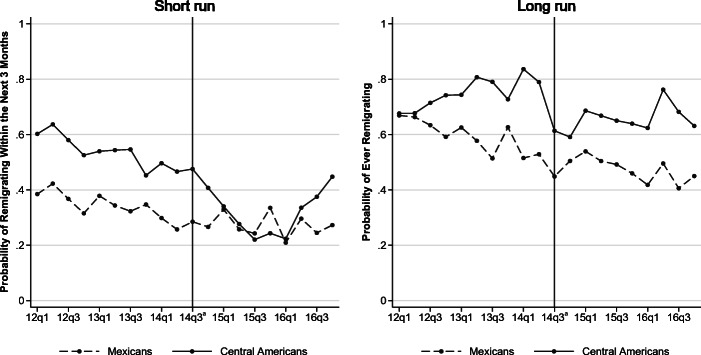
Evolution of the average intentions to remigrate. *Source:* Author’s analysis. ^a^ Quarter when the SBP was introduced.

Figure 3 illustrates the change over time in the share of recidivism intentions for both groups. Two observations stand out. First, reported intentions for both Central American and Mexican deportees followed a similar path before the program was announced, but this is true for only short-run intentions. Although short-run intentions decreased gradually from 2012 to 2014 for both groups, long-run intentions ran in opposite directions. This difference could be explained by budget restrictions (see, e.g., Djajić et al. 2016; Dustmann and Okatenko 2014). Because the trip to the United States is more costly, Central Americans decreased their intentions to migrate in the short run, but the strong push factors (i.e., increasing violence and worsening economic conditions) drove the upward trend in long-run migration intentions.

Second, a clear drop is observed in the share of Central American migrants who report intentions to cross the U.S. border in the short and long run. This drop occurred exactly after July 2014, when the SBP was announced. Short-run migration intentions of Central American deportees dropped to levels lower than those of Mexican deportees after the start of the SBP. In 2016, the remigration intentions started to increase, which is consistent with both the decrease in the number of checkpoints in Mexico (Fig. 2) and the simultaneous increase in apprehensions at the U.S. border (Fig. 1) in the same year. Long-run migration intentions dropped exactly in July 2014, and although the upward trend restarted, the share of migrants reporting intentions to remigrate did not fully catch up with pre-SBP levels. This is the first indication that the SBP had a deterrence effect for the treatment group, but it did not alter the migration intentions of the control group.

The empirical analysis is restricted to short-run remigration intentions because the parallel-trend assumption is clearly violated in the long run. In addition, people’s intentions change over time, so the long-run effect could be substantially different. Individual migration intentions depend not only on known push and pull factors but also on unknown realizations of the future. In the long run, unexpected shocks or specific individual or household characteristics may induce changes in migration costs, thereby fostering, delaying, or preventing the realization of migration plans (Chort 2014).

## Results

### DiD Results

Using the framework in Eq. (1), Table 2 presents the results of a linear probability model on migration intentions. Column 1 presents the results without control variables but including quarter fixed effects and origin department fixed effects. Deportees in the treatment group are 15 percentage points less likely to report intentions to remigrate. With the inclusion of individual characteristics (column 2), trip characteristics (column 3), and apprehension state fixed effects (column 4), the coefficient becomes more negative, and the statistical significance is unchanged. With the inclusion of department-level specific time trends (column 5), the coefficient’s magnitude drops 2 percentage points, suggesting that some factors at the origin-department level diverge for treatment and control units. However, the point estimate remains negative and statistically significant, indicating that these factors are not driving the negative effect of the SBP on migration intentions. The results are similar when controlling for state of apprehension–specific time trends (column 6).

**Table 2:** Effect of Southern Border Plan on the intent to remigrate

	1	2	3	4	5	6
Individual Is Central American × Post-SBP	*−*0*.*148***	*−*0*.*180***	*−*0*.*181***	*−*0*.*182***	*−*0*.*165***	*−*0*.*163***
	(0*.*036)	(0.038)	(0.038)	(0.040)	(0.032)	(0.032)
Male	––	0*.*084***	0*.*084***	0*.*096***	0*.*098***	0*.*098****
		(0*.*022)	(0*.*021)	(0*.*021)	(0*.*020)	(0*.*020)
Age	––	0*.*001	0*.*001	0*.*001*	0*.*001*	0*.*001*
		(0*.*001)	(0*.*001)	(0*.*001)	(0*.*001)	(0*.*001)
Speaks English	––	0*.*046*	0*.*037^†^	0*.*043*	0*.*042^†^	0*.*039^†^
		(0*.*021)	(0*.*021)	(0*.*021)	(0*.*022)	(0*.*023)
Education (ref. = primary or less)						
Secondary education	––	0*.*040**	0*.*043***	0*.*054***	0*.*050***	0*.*051***
		(0*.*012)	(0*.*012)	(0*.*011)	(0*.*011)	(0*.*011)
High school	––	0*.*058*	0*.*058*	0*.*068**	0*.*064**	0*.*062*
		(0*.*024)	(0*.*023)	(0*.*024)	(0*.*024)	(0*.*025)
Tertiary education	––	0*.*106^†^	0*.*114*	0*.*110^†^	0*.*113*	0*.*115*
		(0*.*056)	(0*.*054)	(0*.*057)	(0*.*056)	(0*.*055)
Married	––	–0*.*044***	–0*.*044***	–0*.*042**	–0*.*042**	–0*.*044***
		(0*.*012)	(0*.*012)	(0*.*013)	(0*.*013)	(0*.*013)
Head	––	0*.*037*	0*.*034^†^	0*.*036*	0*.*033^†^	0*.*032^†^
		(0*.*018)	(0*.*017)	(0*.*017)	(0*.*017)	(0*.*017)
Household Size	––	–0*.*002	–0*.*002	0*.*001	0*.*001	0*.*002
		(0*.*004)	(0*.*003)	(0*.*003)	(0*.*003)	(0*.*002)
Has Family/Friends in the United States	––	0*.*106***	0*.*101***	0*.*094***	0*.*088***	0*.*091***
		(0*.*013)	(0*.*013)	(0*.*013)	(0*.*013)	(0*.*013)
Employed Before Migration	––	–0*.*079***	–0*.*083***	–0*.*099***	–0*.*103***	–0*.*107***
		(0*.*017)	(0*.*017)	(0*.*017)	(0*.*017)	(0*.*017)
Ln(Money Spent)	––	––	0*.*007	0*.*008	0*.*020	0*.*024
			(0*.*014)	(0*.*015)	(0*.*015)	(0*.*015)
Borrowed Money to Cross	––	––	0*.*017	0*.*036*	0*.*048**	0*.*047**
			(0*.*016)	(0*.*015)	(0*.*015)	(0*.*015)
Used a Coyote	––	––	–0*.*030	–0*.*029	–0*.*049**	–0*.*056**
			(0*.*020)	(0*.*019)	(0*.*018)	(0*.*018)
People Traveling Together	––	––	0*.*006***	0*.*006***	0*.*006***	0*.*007***
			(0*.*001)	(0*.*001)	(0*.*001)	(0*.*001)
Traveled With Children	––	––	–0*.*046*	–0*.*039^†^	*–*0*.*030	–0*.*028
			(0*.*018)	(0*.*020)	(0*.*020)	(0*.*020)
Previous Number of Crossings	––	––	0*.*011**	0*.*006*	0*.*006*	0*.*005^†^
			(0*.*003)	(0*.*003)	(0*.*003)	(0*.*003)
Constant	0*.*667***	0*.*507***	0*.*476***	0*.*805***	0*.*841***	0*.*600***
	(0*.*024)	(0*.*054)	(0*.*090)	(0*.*095)	(0*.*089)	(0*.*103)
Quarter Fixed Effects	Yes	Yes	Yes	Yes	Yes	Yes
Origin Department Fixed Effects	Yes	Yes	Yes	Yes	Yes	Yes
Apprehension State Fixed Effects	No	No	No	Yes	Yes	Yes
Origin Department Time Trend	No	No	No	No	Yes	Yes
Apprehension State Time Trend	No	No	No	No	No	Yes
Number of Observations	32,041	32,041	32,041	32,041	32,041	32,041
*R*^2^	*.*072	*.*096	*.*100	*.*166	*.*187	*.*193

*Notes:* Results are obtained from OLS regressions. Standard errors, clustered at the origin department level, are shown in parentheses.

^†^*p* < .10; *p < .05; **p < .01; ***p < .001

Together, the results suggest that the SBP curbed crossing intentions among Central American deportees considerably relative to Mexican deportees. The preferred specification (column 6) indicates that Central American deportees are about 16 percentage points less likely to report intentions to remigrate. These results are in line with the theoretical model of Djajić (2017) and are also comparable to the findings in Amuedo-Dorantes and Pozo (2014), who found that deportees apprehended in U.S. states with stricter immigration laws decrease their immediate reentry intentions by 24 percentage points. Thus, migrants seem to respond to policies that aim at checking their immigration status not only in the United States but also while being in transit.

Additional findings from Table 2 show that deportees who are male, have higher levels of education, and have better English skills are more likely to indicate intentions to remigrate. Deportees with relatives or friends in the United States are also more likely to report intentions to remigrate, highlighting the relevance of networks in the destination country on the decision to migrate (McKenzie and Rapoport 2007). In contrast, deportees who are married or were employed in the origin country before migration are less likely to report recidivism intentions. These findings are not surprising: they reflect characteristics of migrants who are more attached to their origin country. A surprising result is that deportees who borrowed money for their trip are more likely to indicate reentry intentions. Although this contradicts the findings in Amuedo-Dorantes et al. (2015), a plausible explanation is that migrants who borrowed money in the past might be able to borrow again in the future to finance the costs of another trip.

### Robustness Checks

To test the causal interpretation of the results reported in the previous subsection, I conduct several robustness tests and report the results in Table 3. To discard that possible omitted time-varying factors are driving the results, I test whether the intention to remigrate is correlated with a placebo program. During the pre-program period—from January 2012 to June 2014—none of the migrants were exposed to additional enforcement from Mexican authorities. I conduct a falsification experiment by restricting the sample to individuals who crossed the border during the pre-program period. I create a false program in the same quarter of the year when the SBP was announced by including a binary variable that takes the value of 1 from the third quarter of 2012 onward. I then reestimate the specification presented in Eq. (1). The experiment is also conducted for the third quarter of 2013 onward. A statistically significant β would indicate that underlying factors correlated with the SBP are driving the results. The point estimates are shown in panel A and correspond to the specifications in Table 2 (columns 1–6). In both experiments, the coefficients are close to 0 and not statistically significant in any of the specifications, indicating that the estimated effect is not driven by pre-program group differences.

**Table 3:** Effect of Southern Border Plan on the intent to remigrate: Robustness

	1	2	3	4	5	6
A. Placebo Experiment						
Placebo program (Q3, 2012)						
Individual is Central American × post-SBP	−0*.*018	−0*.*001	0*.*001	0*.*007	0*.*003	−0*.*004
	(0*.*030)	(0*.*029)	(0*.*028)	(0*.*028)	(0*.*044)	(0*.*043)
Number of observations	18,462					
Placebo program (Q3, 2013)						
Individual is Central American × post-SBP	−0*.*016	−0*.*005	−0*.*001	0*.*013	0*.*002	0*.*004
	(0*.*039)	(0*.*040)	(0*.*038)	(0*.*037)	(0*.*049)	(0*.*049)
Number of observations	18,462					
B. Alternative Post-Program Period: 2012–2016 (excluding Q3 and Q4 of 2014)						
Individual is Central American × post-SBP	−0.165***	−0.202***	−0.204***	−0.204***	−0.263***	−0.263***
	(0.039)	(0.040)	(0.041)	(0.040)	(0.049)	(0.049)
Number of observations	29,568					
C. By Origin Country						
El Salvador						
Individual is Central American × post-SBP	−0.413***	−0.446***	−0.462***	−0.468***	−0.222***	−0.226***
	(0*.*021)	(0*.*022)	(0*.*028)	(0*.*025)	(0*.*035)	(0.035)
Number of observations	21,175					
Guatemala						
Individual is Central American × post-SBP	0*.*074**	0*.*051*	0*.*049^†^	0*.*038	–0*.*093*	–0*.*089*
	(0*.*025)	(0*.*025)	(0*.*025)	(0*.*024)	(0*.*037)	(0*.*040)
Number of observations	15,714					
Honduras						
Individual is Central American × post-SBP	–0*.*124***	–0*.*169***	–0*.*163***	–0*.*146***	–0*.*165***	–0*.*160***
	(0*.*029)	(0*.*031)	(0*.*030)	(0*.*030)	(0*.*038)	(0*.*038)
Number of observations	14,530					
D. U.S. Border Patrol Sector Fixed Effects						
Individual is Central American × post-SBP	–0.109**	–0.143***	–0.145***	–0.131***	–0.121***	–0.120***
	(0*.*037)	(0*.*038)	(0*.*039)	(0*.*038)	(0*.*032)	(0*.*032)
Number of observations	29,291					
E. State of Origin–Apprehension Fixed Effects^a^						
Individual is Central American × post-SBP	––	––	––	–0*.*151***	–0*.*154***	–0*.*151***
				(0*.*030)	(0*.*042)	(0*.*042)
Number of observations	32,041					

*Notes:* Results are obtained from OLS regressions. Each column controls for the same variables as in Table 2. Standard errors, clustered at the origin department level, are shown in parentheses.

^a^Controls for state of origin–apprehension fixed effects. The standard errors are clustered at the state of origin-apprehension level.

^†^*p* < .10; *p < .05; **p < .01; ***p < .001

To show that the results are not driven by observations right after the SBP was announced, I redefine the post-program binary indicator by shifting the treatment to the first quarter of 2015. The variable *PostSBP* in Eq. (1) now equals 1 for deportees who started their trip in 2015, and 0 otherwise. All migrants who started their trip in the third and fourth quarter of 2014 are dropped from the sample. Panel B of Table 3 shows that the results are robust to the alternative definition of the post-program period.

I conduct the analysis for each origin country separately to show that the results are not driven by one origin country in particular. Panel C reveals that the size of the effect varies across countries but is negative and statistically significant for the preferred specification in column 6 for all countries.

The estimation may not take into account enforcement measures at the U.S.-Mexico border and how these affect migration intentions of deportees. The analyses so far have assumed that changes in migration enforcement at the U.S. border are a common shock for both treatment and control groups and should be accounted for by including time fixed effects. This assumption is plausible if Mexicans and Central Americans choose the same locations to cross the U.S.-Mexico border. If this is not the case, enforcement changes in U.S. BP sectors would affect both groups differently.^20^ To test this empirically, I control for BP sector fixed effects instead of apprehension state fixed effects. Mexican deportees indicated the Mexican border city where they crossed the border, and Central American deportees indicated the city they were heading to (to cross the U.S.-Mexico border). Thus, I assign a BP sector to both groups of deportees. The BP fixed effects capture time-invariant characteristics that correlate with the decision to migrate, such as specific geographic characteristics at the border or sectors with stricter enforcement measures in place. The results reported in panel D of Table 3 are robust to the inclusion of BP sector fixed effects and the interaction of the fixed effects with a linear time trend.

The baseline specification does not take into account that distance between the migrant’s origin department and state of apprehension may be a determinant of migration intentions. I include state of origin–apprehension dyadic fixed effects instead of the additive fixed effects. State of origin–apprehension fixed effects control for time-invariant bilateral characteristics, such as distance. The coefficients reported in Panel E of Table 3 are slightly larger than the baseline specification and remain statistically significant.

Two main concerns arise about the validity of the control group. First, Mexicans could be indirectly affected by the SBP if, for example, given that fewer Central American migrants are arriving at the U.S. border, Mexicans are more likely to be apprehended. Nevertheless, U.S. BP data show that apprehensions of Mexicans did not spike despite the large decrease in apprehensions of Central Americans, and the share of Mexican deportees reporting intentions to remigrate in the short run remains unchanged (see Fig. 3).^21^ Second, time-varying confounders could drive migration intentions in the treatment group. For example, increasing push factors in the Northern Triangle have driven more Central American migrants to seek asylum in the United States.

To address these concerns, I follow an approach similar to that in Duflo (2001) to define an alternative control group based on the timing of migration. I restrict the sample to Central American deportees apprehended in Mexico between 2013 and 2014. The control group is now defined by Central American deportees who started their trip sometime between January and December 2013, and the treatment group by Central American deportees who started their trip sometime between January and December 2014. The policy variable is equal to 1 if the migrant started the trip in the second half of the year (between July and December) to account for the introduction of the SBP. The estimated DiD coefficient shows the difference in intentions of the cohort exposed to the program (Central Americans who migrated in 2014) relative to that of the cohort not exposed to the program (Central Americans who migrated in 2013). The estimated coefficients reported in Table 4 are smaller but remain negative and statistically significant.

**Table 4:** Effect of Southern Border Plan on the intent to remigrate: Alternative control group

	1	2	3	4	5	6
Trip 2014 × Trip Q3–Q4	−0.074***	−0.094***	−0.100***	−0.102***	−0.104***	−0.109***
	(0.016)	(0.017)	(0.016)	(0.016)	(0.017)	(0.017)
Male	––	0.050^†^	0.087***	0.090***	0.087***	0.086***
		(0.027)	(0.021)	(0.021)	(0.021)	(0.022)
Age	––	−0.001	−0.000	−0.000	−0.000	−0.000
		(0.001)	(0.001)	(0.001)	(0.001)	(0.001)
Speaks English	––	−0.010	−0.018	−0.023	−0.004	0.004
		(0.084)	(0.090)	(0.089)	(0.093)	(0.096)
Education (ref. = primary or less)						
Secondary education	––	–0.004	–0.003	−0.003	0.003	0.004
		(0.013)	(0.013)	(0.013)	(0.013)	(0.012)
High school	––	−0.028	−0.030^†^	−0.029	−0.026	−0.024
		(0.018)	(0.017)	(0.018)	(0.018)	(0.018)
Tertiary education	––	−0.017	−0.018	−0.014	−0.004	−0.007
		(0.051)	(0.048)	(0.048)	(0.046)	(0.046)
Married	––	−0.024	−0.029	−0.031	−0.032	−0.032
		(0.023)	(0.024)	(0.024)	(0.024)	(0.024)
Head	––	0.008	0.017	0.017	0.014	0.015
		(0.021)	(0.023)	(0.023)	(0*.*023)	(0.023)
Household Size	––	0.001	0.003	0.003	0.002	0.002
		(0.003)	(0.003)	(0.003)	(0.003)	(0.003)
Has Family/Friends in the United States	––	0.026	0.020	0.022	0.021	0.021
		(0.018)	(0.016)	(0.016)	(0.017)	(0.017)
Employed Before Migration	––	–0.090***	–0.069**	–0.071***	–0.072***	–0.073***
		(0.019)	(0.020)	(0.020)	(0.020)	(0.020)
Ln(Money Spent)	––	––	0.021	0.024	0.031*	0.031*
			(0.014)	(0.015)	(0.014)	(0.014)
Borrowed Money to Cross	––	––	0.168***	0.165***	0.162***	0.163***
			(0.021)	(0.021)	(0.021)	(0.021)
Used a Coyote	––	––	0.041^†^	0.038^†^	0.011	0.011
			(0.022)	(0.022)	(0.021)	(0.021)
People Traveling Together	––	––	0.017***	0.017***	0.017***	0.017***
			(0.004)	(0.004)	(0.004)	(0.004)
Traveled With Children	––	––	–0.106**	–0.105**	–0.099**	–0.101**
			(0.035)	(0.035)	(0*.*034)	(0.035)
Previous Number of Crossings	––	––	–0.035^†^	–0.033	–0.035	–0.035
			(0.020)	(0.021)	(0*.*021)	(0.021)
Constant	0.631***	0.663***	0.390***	0.382*	0.403**	0.262
	(0.012)	(0.045)	(0.087)	(0.145)	(0.141)	(0.160)
Quarter Fixed Effects	Yes	Yes	Yes	Yes	Yes	Yes
Origin Department Fixed Effects	Yes	Yes	Yes	Yes	Yes	Yes
Apprehension State Fixed Effects	No	No	No	Yes	Yes	Yes
Origin Department Time Trend	No	No	No	No	Yes	Yes
Apprehension State Time Trend	No	No	No	No	No	Yes
Number of Observations	10,099	10,099	10,099	10,099	10,099	10,099
*R*^2^	*.*086	*.*093	*.*128	*.*131	*.*145	*.*148

*Notes:* Results are obtained from OLS regressions. The treatment group is defined as deportees originating from El Salvador, Honduras, and Guatemala who started their trip sometime between January and December 2014. The control group is defined as deportees who started their trip sometime between January and December 2013. Standard errors, clustered at the origin department level, are shown in parentheses.

^†^*p* < .10; *p < .05; **p < .01; ***p < .001

Finally, to show that the SBP not only had an impact on migration intentions but also on observed migration outcomes, I conduct an exercise using data on monthly apprehensions at the U.S. border as a proxy for unauthorized migration flows. Although this exercise is informative on how apprehensions in the United States changed after the SBP was implemented, they must be interpreted with caution given that apprehensions are a highly imperfect proxy for migration flows.

I run a DiD regression using data on monthly apprehensions of Central Americans (treatment group) and Mexican unauthorized immigrants (control group) by U.S. BP sector. Table 5 reports the results. Column 1 controls for country of origin, BP sector, year fixed effects, and a group-specific linear time trend. Column 2 further includes month fixed effects to capture the seasonal variation. The estimated coefficients reflect the change on apprehensions of Central American unauthorized migrants relative to Mexican unauthorized migrants right after the SBP was introduced. The results show a negative and significant effect of the SBP on apprehensions of Central American migrants at the U.S. border of about 488 apprehensions by BP, which translates to a decrease in monthly apprehensions of unauthorized Central Americans at the U.S. border by about 5,000. Columns 3 and 4 present the results of introducing a placebo program in the third quarter of 2013 and are not statistically significant, thus indicating that the effects in columns 1 and 2 are not exclusively driven by pre-program differences between the groups. These results show that the SBP leads to a reduction not only in migration intentions but also in apprehensions of Central American migrants at the U.S. border.

**Table 5:** Effect of Southern Border Plan on aggregate apprehensions by the U.S. Border Patrol

	DiD Estimates		Placebo Estimates	
	1	2	3	4
Central American Apprehensions × Post-SBP	−555.862***	−488.250***	46.481	178.605
	(143.619)	(137.474)	(97.474)	(115.526)
Country of Origin Fixed Effects	Yes	Yes	Yes	Yes
Border Patrol Sector Fixed Effects	Yes	Yes	Yes	Yes
Year Fixed Effects	Yes	Yes	Yes	Yes
Country of Origin Time Trends	Yes	Yes	Yes	Yes
Month Fixed Effects	No	Yes	No	Yes
Number of Observations	2,160	2,160	864	864
*R*^2^	.613	.618	.588	.596

*Notes:* Results are obtained from OLS regressions. The data refer to monthly apprehensions by Border Patrol sector of Central Americans and Mexicans. The treatment group is defined as apprehensions of unauthorized migrants originating from El Salvador, Honduras, and Guatemala. The control group is defined as the monthly apprehensions of unauthorized Mexicans. For columns 1 and 2, the pre-program period is defined as the interval between January 1, 2012 and June 30, 2014. For the placebo test in columns 3 and 4, the pre-program period is defined as the interval between January 1, 2012 and June 30, 2013. Robust standard errors are shown in parentheses.

***p < .001

The number of apprehensions by the U.S. BP is equal to the probability of being apprehended multiplied by the number of crossings. The SBP could have reduced apprehensions through either or both of these factors. The probability of being apprehended during a crossing is a positive function of the BP skill and a negative function of the migrant’s evasion skills. The SBP could have decreased the probability of apprehension if only very skilled evaders are the ones who decide to remigrate. Alternatively, the SBP could have decreased the number of crossings if the SBP is effective at intercepting migrants who are in transit or if it deterred actual attempts. The findings from Tables 2 and 5 combined suggest that the estimated negative effect of the SBP on migration intentions is not purely driven by changes in the sample composition and that remigration attempts likely decreased.

### Heterogeneous Effects

**Fig. 4:**
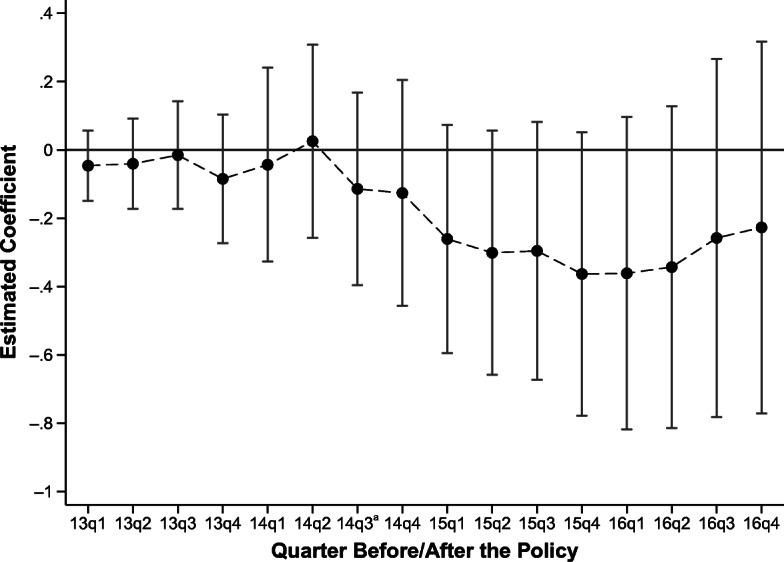
Estimated impact of the Southern Border Plan on migration intentions. The results are obtained from ordinary least squares (OLS) regressions of Eq. (1), including the full set of control variables, state of origin, state of apprehension, and time fixed effects, as well as an interaction with a linear time trend. Confidence intervals are calculated at the 95% level, and the standard errors are clustered at the origin department level. The parameters for the quarters 14q3–16q4 are jointly statistically significant at the 95% level of confidence. ^a^ Quarter when the SBP was introduced.

To test whether deportees respond to the program with a time lag and whether this response is time-persistent, I reestimate Eq. (1) to include interaction terms for the treatment group with quarter dummy variables. Figure 4 plots the point estimates and their respective confidence intervals. Although all the point estimates before the third quarter of 2014 (pre-program period) are almost equal to 0, negative point estimates can be observed in the post-program period. Although the point estimates are not statistically significant, they are jointly significant at the 5% level. The estimates become more negative after the first quarter of 2015, which is consistent with the descriptive evidence provided in Fig. 2 showing that the largest increase in checkpoints and raids by Mexican authorities started after January 2015.

To test heterogeneous effects for different subpopulation groups, I interact the treatment effect with a categorical variable that indicates whether the migrant has access to a network in the United States and the migrants’ (1) gender, (2) migration experience, and (3) employment status before migration, respectively.^22^ The results are reported in Table 6. Focusing on gender differences, column 1 shows that for both men and women, the SBP’s deterrence effect is larger for individuals without a network in the United States, but the difference between groups is significant only for men (column 2). Men without a network in the United States are 21 percentage points less likely to indicate intentions to remigrate, and the coefficient for men with access to a network is almost one-half that size, at 11 percentage points.

**Table 6:** Heterogeneous effects of Southern Border Plan on the intent to remigrate

	Gender		Migration Experience		Employment Status	
	1	2	3	4	5	6
SBP Effect × Network, Male	–0*.*114*					
	(0*.*045)					
SBP Effect × Network, Female	–0*.*134^†^	[0*.*758]				
	(0*.*068)					
SBP Effect × No Network, Male	–0*.*214***	[0*.*042]				
	(0*.*038)					
SBP Effect × No Network, Female	–0*.*197^†^	[0*.*519]				
	(0*.*118)					
SBP Effect × Network, Experience			–0*.*106^†^			
			(0*.*062)			
SBP Effect × Network, No Experience			–0*.*143**	[0*.*466]		
			(0*.*044)			
SBP Effect × No Network, Experience			–0*.*318***	[0*.*011]		
			(0*.*062)			
SBP Effect × No Network, No Experience			–0*.*221***	[0*.*049]		
			(0*.*048)			
SBP Effect × Network, Employed					–0*.*171***	
					(0*.*046)	
SBP Effect × Network, Unemployed					–0*.*068	[0*.*028]
					(0*.*055)	
SBP Effect × No Network, Employed					–0*.*170***	[0*.*996]
					(0*.*038)	
SBP Effect × No Network, Unemployed					–0*.*293***	[0*.*106]
					(0*.*068)	

*Notes:* Results are obtained from OLS regressions. The total number of observations for each regression is 32,041. The regressions include the full set of control variables as in column 6 of Table 2, and the interaction of the respective group indicators with the (1) treatment effect, (2) time fixed effects, and (3) origin department fixed effects. Standard errors are in parentheses (clustered at the origin department level). The figures in squared brackets correspond to the *p* value for the test that the estimated coefficient is the same as the first coefficient in the group.

^†^*p* < .10; *p < .05; **p < .01; ***p < .001

Focusing on migration experience, column 3 shows a similar pattern. Irrespective of migration experience, the deterrence effect is larger for migrants without access to a network, and the difference between the coefficients and the reference group is statistically significant (column 4). Finally, focusing on the pre-migration employment indicator, column 5 shows that irrespective of having access to a network, deportees who were employed before migration responded to the SBP in a similar way. The same is true for unemployed migrants without access to a network. For these groups, I find no statistically significant differences (column 6). In contrast, migrants who were unemployed but had access to a network were not deterred by the SBP. The estimated coefficient is close to 0 and not statistically significant. These findings suggest that even if individuals find it optimal to migrate, budget constraints may not allow them to cover the cost of migration (see, e.g., Djajić et al. 2016; Dustmann and Okatenko 2014), and having a network in the United States appears to alleviate some of these constraints.

Taken together, the results in Table 6 suggest that migrants with access to a network in the United States are less deterred by the SBP, which relates to the literature on cumulative causation^23^ and migration networks. The cumulative causation literature shows that as community members gain migration-related knowledge through family members and friends in the destination country, the likelihood to migrate increases for other members of the community (Curran and Rivero-Fuentes 2003; Fussell and Massey 2004). The literature on migration networks has shown that networks can (1) be a source of credit (Comola and Mendola 2015), (2) ease the access to the labor market, and (3) improve labor market opportunities for the migrant through, for example, the transition from agricultural to nonagricultural jobs (Munshi 2003). Family networks of undocumented migrants encourage both migration and coyote use, and provide information on border crossing and ways to procure a smuggler (Dolfin and Genicot 2010; McKenzie and Rapoport 2010).

I further test whether the SBP curbs recidivism intents of the group of migrants not apprehended and deported by Mexican authorities. I estimate Eq. (1) using data on Central American migrants apprehended and deported by U.S. authorities (EMIF South). The data consist of Central American deportees who started their trip to the United States between 2012 and 2016 and transited through Mexico. The main difference is that these migrants were caught by U.S. authorities but not Mexican authorities while in transit. The results shown in Table 7 provide evidence on how irregular immigrants respond to tougher enforcement if they are not apprehended, even if they cannot be generalized to migrants who settled in the United States illegally. The effect of the SBP on the probability of reporting intentions to remigrate is essentially 0 for the group of deportees apprehended by U.S. authorities, implying that additional enforcement in Mexico is effective in deterring migrants only if they are apprehended and deported while in transit.

**Table 7:** Effect of Southern Border Plan on the intent to remigrate: Deported by U.S. authorities

	1	2	3	4	5	6
Individual Is Central American × Post-SBP	0*.*050	0*.*035	0*.*037	0*.*044	–0*.*017	–0*.*014
	(0*.*021)	(0*.*023)	(0*.*024)	(0*.*022)	(0*.*039)	(0*.*038)
Quarter Fixed Effects	Yes	Yes	Yes	Yes	Yes	Yes
Origin Department Fixed Effects	Yes	Yes	Yes	Yes	Yes	Yes
U.S. State Fixed Effects	No	No	No	Yes	Yes	Yes
Origin Department Time Trend	No	No	No	No	Yes	Yes
U.S. State Time Trend	No	No	No	No	No	Yes
Number of Observations	27,458	27,458	27,456	27,456	27,456	27,456
*R*^2^	*.*060	*.*079	*.*084	*.*114	*.*122	*.*126

*Notes:* Results are obtained from OLS regressions. Each column controls for the same variables as in Table 2. Standard errors, clustered at the origin department level, are shown in parentheses.

On the reasons why the SBP has no effect on the remigration intentions of deportees apprehended in the United States, I can only hypothesize. This group of migrants did not actually experience apprehension in Mexico and are thus less responsive to the SBP. The crime literature on deterrence and the reasons why some individuals are more responsive than others to the threat of punishment supports this argument.^24^ Crime deterrence involves several factors, such as how well informed the individual is on the threat, the severity of the sanctions, and the individual’s perceived risk of being apprehended (Apel 2013). Risk perceptions are sensitive to actual experience. Anwar and Loughran (2011) found that individuals who commit a crime and are apprehended increase their perceived probability of being caught compared with individuals who were not apprehended. Thus, the insignificant effect for deportees apprehended in the United States could be explained by their actual experience in Mexico. Given that they were not apprehended, their perceived probability of apprehension did not increase to the level of deportees who were apprehended.

## Conclusion

In this study, I examine whether the SBP, an enforcement initiative announced by the Mexican government to deter unauthorized migrants in transit through Mexico, has curbed the remigration intentions of Central American deportees. Using a DiD approach, I estimate the effect of the SBP on the intention to remigrate for Central American migrants in transit to the United States (apprehended by Mexican authorities) relative to Mexican deportees (apprehended by U.S. authorities).

The results reveal that the introduction of the SBP decreases the likelihood of reporting recidivism intentions by 16 percentage points. Before the SBP plan was introduced, 54% of Central American deportees stated their intention to remigrate in the short run. These measures combine to suggest that the SBP decreased migration intentions by 30%, which is a sizable effect. Using data on apprehensions at the U.S. BP sector, I show that monthly apprehensions of Central Americans relative to Mexicans decreased, on average, by about 500 by BP sector after the SBP was introduced. Taken together, these results suggest that migration attempts likely decreased after the introduction of the SBP. The results are robust to a number of specifications and to the definition of an alternative control group.

The most important source of heterogeneity of the SBP’s effect on remigration intentions is having access to a network in the United States. Migrants with family or friends in the United States are less deterred by the SBP, highlighting the importance of access to networks for the decision to migrate (see, e.g., Dolfin and Genicot 2010). For instance, even if migrants are budget constrained, they do not respond to the SBP if they have access to a network.

Although the external validity of my findings to other countries and migration routes remains an open question, they strongly support the argument of Djajić (2017) that migration enforcement in transit countries can reshape migration intentions in the short run. The success of immigration enforcement efforts in reducing Central American unauthorized migration in the United States depends not only on the actions taken by U.S. immigration authorities but also on Mexico’s immigration policy. This result is particularly relevant given the surge in undocumented migration from Central America to the United States in the last decade. Yet, given the vulnerability of migrants in transit and evidence that migration enforcement is not without unintended consequences (see, e.g., Amuedo-Dorantes and Pozo 2014), increased enforcement in Mexico should not be a substitute for safe and legal migration channels.

## Electronic supplementary material

ESM 1 (PDF 380 kb)

## Data Availability

The data sets generated and/or analyzed during the current study are available from the corresponding author on reasonable request.

## Footnotes

1 The IOM estimates the number of international migrants globally at about 244 million.

2 Data on apprehensions are a highly imperfect proxy for the total flow of undocumented migrants because an undocumented migrant can be caught multiple times during the same month, but other migrants are not apprehended (Cornelius 2001). Nevertheless, data on apprehensions can shed some light on the number of arrivals and the flow composition (Hanson 2006).

3 The first sought to increase investments in Libya’s border controls and sea surveillance to deter African immigrants. The second aimed to close the Balkan route, reducing migration flows in transit through Turkey to Greece, prevent new sea or land routes, and establish relocation agreements (European Commission 2017).

4 A notable exception is the work by Carling (2007), who analyzed the effect of migration controls on Spanish-African borders on the number of deaths.

5 An exception is Guido et al. (2017), who focused on the impact of rising smuggling markets in transit countries on migration intentions. However, this study differs from mine because it did not analyze immigration enforcement directly.

6 For literature reviews on irregular migration from Mexico to the United States, see Hanson (2006) and Orrenius (2014).

7 Very few migrants from the Northern Triangle enter the United States legally and then overstay their visa. Obtaining a visa is difficult if the applicant does not meet the eligibility requirements, such as having assets or being employed in the country of origin. The overstay rate for individuals from Guatemala, Honduras, and El Salvador admitted to the United States via air or sea for business or pleasure (B1/B2 visa) is small at 2.20%, 2.39%, and 2.65%, respectively (U.S. Department of Homeland Security (DHS) 2016). The DHS estimates the overstay rate using information on arrivals and departures. Due to limitations in collecting departure data, the overstay rate is an estimate and should be interpreted with caution.

8 This initiative is a package of U.S. assistance for Mexico targeted at four pillars: (1) disrupting criminal groups, (2) institutionalizing the rule of law, (3) creating a twenty-first century border, and (4) building strong and resilient communities.

9 Rodríguez Chávez (2017) calculated annual average flows using apprehensions both in Mexico and the United States as well as an estimated number of individuals who were not apprehended and managed to settle in the United States.

10 The SBP is also referred to as Southern Border Program (*Programa Frontera Sur*).

11 These types of policies are highly controversial because they create a basis for racial profiling and discrimination (Hoekstra and Orozco-Aleman 2017).

12 Department is an aggregate measure equivalent to states.

13 In the United States, the number of affirmative asylum applications from individuals originating from the Northern Triangle increased from 7,723 to 25,801 between 2014 and 2016 (Mossaad and Baugh 2016). In Mexico, asylum applications increased from 1,772 to 8,060 over the same period (Comisión Mexicana de Ayuda a Refugiados (COMAR) 2016).

14 These organizations are *Secretaría de Gobernación* (SEGOB), *Unidad de Política Migratoria* (IPM), *Consejo Nacional de Población* (CONAPO), *Consejo Nacional para Prevenir la Discriminación* (CONAPRED), *Secretaría de Relaciones Exteriores* (SRE), *Secretaría del Trabajo y Prevención Social* (STPS), and *Secretaría de Desarrollo Social* (SEDESOL).

15 These data have been used by Amuedo-Dorantes and Pozo (2014), Amuedo-Dorantes and Lozano (2015), and Moraga (2011).

16 This is an advantage over the Current Population Survey (CPS) and the American Community Survey (ACS), which would allow only estimating the impact of the SBP on population changes in the United States of a subset of Hispanics who are likely unauthorized.

17 The exact date when the individual started the trip is not provided. For Central Americans, I calculate the date using the date of apprehension in Mexico minus the days spent traveling. Because for Mexicans time spent traveling is not available, I calculate the starting date using the survey date minus the number of days spent in the United States and the days spent at the Mexican border.

18 In 2013, the stock of emigrants as percentage of the population was 25%, 6.7%, and 8.4% for El Salvador, Guatemala, and Honduras, respectively (Ratha et al. 2016).

19 Tables with descriptive statistics for the full sample are reported in Tables A1 and A2 in the online appendix.

20 Data on apprehensions from the U.S. Department of Homeland Security show that the share of apprehensions of Central American migrants relative to Mexican migrants increased in all U.S. BP sectors from 2012 to 2015. The majority of apprehensions for both groups happens in the sectors Rio Grande Valley (Texas), Tucson (Arizona), and Laredo (Texas).

21 I test whether the reported migration intentions of Mexican and Central American migrants are correlated with the introduction of the SBP by running a linear regression for each group separately. Although migration intentions of Central American deportees are strongly negatively correlated with the introduction of the SBP, the estimated coefficients for Mexican deportees are not statistically different from 0 in any of the specifications. The results are available upon request.

22 In Table A4 in the online appendix, I show the results of interacting the treatment effect with each group separately. The results show no significant differences except when looking at access to a network in the United States. Migrants without a network in the United States are 23 percentage points less likely to indicate intentions to remigrate, and those with access to a network are 12 percentage points less likely.

23 The literature on cumulative causation explains why migration flows, once they start, continue to grow and become self-sustaining (Massey et al. 1993).

24 See Chalfin and McCrary (2017) for a review of the literature on crime and deterrence.
