# Supplementary material for: The Effects of Enhanced Enforcement at Mexico’s Southern Border: Evidence From Central American Deportees
Source: Demography. 2020 Sep 10;57(5):1597–623. doi: 10.1007/s13524-020-00914-3 (PMC7584557; doi:10.1007/s13524-020-00914-3)
Supplement: Supplementary file 1 — (PDF 380 kb) [file 13524_2020_914_MOESM1_ESM.pdf]

Online Appendix

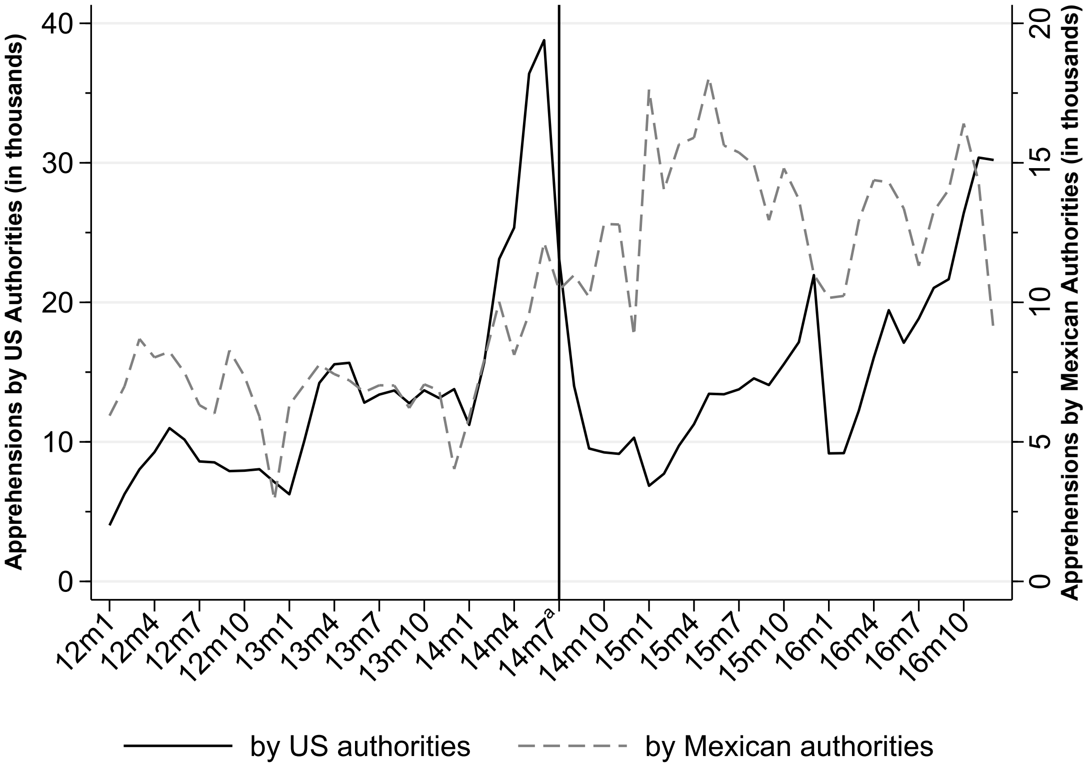

Fig. A1: Monthly apprehensions of undocumented Central Americans in the US and Mexico

Source: Author's analysis using data requested from the INM and US BP.
Note: – [a] Month during which SBP was introduced.

Table A1: Descriptive Statistics: Central American deportees – by Mexican authorities

	All deportees		Pre-Plan		Post-Plan		
	Mean	S.D.	Mean	S.D.	Mean	S.D.	Δ Mean
**Dependent variables**							
Intent to remigrate (short run)	0.423	0.494	0.537	0.499	0.335	0.472	-0.202^{***}
Intent to ever remigrate	0.704	0.457	0.756	0.430	0.663	0.473	-0.093^{***}
**Independent variables**							
Male	0.828	0.378	0.862	0.345	0.801	0.399	-0.061^{***}
Age	27.431	7.688	27.104	7.172	27.687	8.060	0.583^{***}
Speaks English	0.010	0.100	0.006	0.079	0.013	0.113	0.007^{***}
Education							
Primary education or less	0.584	0.493	0.637	0.481	0.543	0.498	-0.094^{***}
Secondary education	0.241	0.427	0.210	0.407	0.264	0.441	0.054^{***}
High school	0.163	0.369	0.143	0.350	0.178	0.383	0.035^{***}
Tertiary education	0.013	0.113	0.010	0.101	0.015	0.121	0.005^{***}
Married	0.422	0.494	0.410	0.492	0.432	0.495	0.022^{*}
Head	0.412	0.492	0.409	0.492	0.414	0.493	0.005
HH size	5.294	2.208	5.571	2.108	5.078	2.259	-0.493^{***}
Employed before migration	0.551	0.497	0.679	0.467	0.451	0.498	-0.227^{***}
Has family/friends in the U.S.	0.699	0.459	0.608	0.488	0.770	0.421	0.162^{***}
Money spent (in 1,000 USD)[a]	0.594	1.049	0.560	1.031	0.621	1.063	0.062^{***}
Borrowed money to cross	0.403	0.491	0.369	0.482	0.431	0.495	0.062^{***}
Used a coyote	0.151	0.358	0.179	0.383	0.128	0.335	-0.050^{***}
People traveling together	0.852	1.829	1.202	2.005	0.578	1.628	-0.624^{***}
Traveled with children	0.047	0.212	0.030	0.171	0.060	0.238	0.030^{***}
Previous number of crossings	0.121	0.421	0.152	0.437	0.096	0.406	-0.055^{***}
Country of origin							
El Salvador	0.275	0.447	0.257	0.437	0.289	0.454	–
Guatemala	0.301	0.459	0.305	0.460	0.299	0.458	–
Honduras	0.423	0.494	0.438	0.496	0.412	0.492	–
Observations	22,352		12,172		10,180		

Notes: – [a] *Calculated using predicted travel costs plus the reported smuggler fees. – Pre-program defined as the interval between 1/1/2012 to 30/6/2014. Post-program defined as the interval between 1/7/2014 and 31/12/2016. –* *** $p < 0.001$; ** $p < 0.01$; * $p < 0.05$; † $p < 0.1$.

Table A2: Descriptive Statistics: Mexican deportees

	All deportees		Pre-Plan		Post-Plan		
	Mean	S.D.	Mean	S.D.	Mean	S.D.	Δ Mean
**Dependent variables**							
Intent to remigrate (short run)	0.335	0.472	0.362	0.481	0.273	0.446	−0.089***
Intent to ever remigrate	0.572	0.495	0.615	0.487	0.474	0.499	−0.141***
**Independent variables**							
Male	0.888	0.315	0.870	0.336	0.930	0.255	0.060***
Age	29.467	8.353	29.253	8.277	29.961	8.506	0.708**
Speaks English	0.126	0.332	0.136	0.343	0.103	0.304	−0.034**
Education							
Primary education or less	0.304	0.460	0.303	0.460	0.305	0.460	0.002
Secondary education	0.505	0.500	0.493	0.500	0.532	0.499	0.039*
High school	0.170	0.376	0.181	0.385	0.146	0.354	−0.034**
Tertiary education	0.021	0.145	0.023	0.151	0.017	0.129	−0.006
Married	0.565	0.496	0.572	0.495	0.549	0.498	−0.023
Head	0.571	0.495	0.582	0.493	0.545	0.498	−0.037*
HH size	5.029	2.506	4.816	2.155	5.521	3.116	0.704***
Employed before migration	0.537	0.499	0.533	0.499	0.544	0.498	0.011
Has family/friends in the U.S.	0.639	0.480	0.635	0.481	0.647	0.478	0.011
Money spent (in 1,000 USD)^a	2.166	2.167	1.967	1.818	2.625	2.757	0.658***
Borrowed money to cross	0.732	0.443	0.733	0.442	0.731	0.443	−0.002
Used a coyote	0.573	0.495	0.586	0.493	0.543	0.498	−0.043**
People traveling together	3.122	5.094	3.553	5.633	2.129	3.345	−1.424***
Traveled with children	0.038	0.192	0.045	0.206	0.025	0.155	−0.020***
Previous number of crossings	1.064	2.314	1.026	2.256	1.152	2.442	0.126†
Observations	9,689		6,290		3,399		

*Notes: – ^{a} Calculated using predicted travel costs plus the reported smuggler fees. – Pre-program defined as the interval between 1/1/2012 to 30/6/2014. Post-program defined as the interval between 1/7/2014 and 31/12/2016. – *** $p < 0.001$; ** $p < 0.01$; * $p < 0.05$; † $p < 0.1$.*

Table A3: Descriptive Statistics: Central American deportees – by US authorities

	All deportees		Pre-Plan		Post-Plan		
	Mean	S.D.	Mean	S.D.	Mean	S.D.	Δ Mean
**Dependent variables**							
Intent to remigrate (short run)	0.147	0.354	0.146	0.353	0.149	0.356	0.004
Intent to ever remigrate	0.501	0.500	0.481	0.500	0.534	0.499	0.053***
**Independent variables**							
Male	0.874	0.331	0.885	0.320	0.857	0.350	−0.027***
Age	27.337	7.878	27.392	7.879	27.242	7.875	−0.150
Speaks English	0.089	0.285	0.087	0.281	0.094	0.292	0.007
Education							
Primary education or less	0.052	0.221	0.058	0.233	0.041	0.199	−0.017***
Secondary education	0.360	0.480	0.391	0.488	0.308	0.462	−0.082***
High school	0.308	0.462	0.308	0.462	0.309	0.462	0.001
Tertiary education	0.251	0.434	0.220	0.414	0.304	0.460	0.084***
Married	0.443	0.497	0.481	0.500	0.378	0.485	−0.103***
Head	0.473	0.499	0.500	0.500	0.426	0.494	−0.075***
HH size	4.823	2.346	4.954	2.360	4.599	2.306	−0.355***
Employed before migration	0.462	0.499	0.566	0.496	0.286	0.452	−0.280***
Has family/friends in the U.S.	0.848	0.359	0.828	0.377	0.880	0.325	0.052***
Money spent (in 1,000 USD)	3.338	1.629	2.995	1.532	3.921	1.624	0.925***
Borrowed money to cross	0.738	0.440	0.726	0.446	0.758	0.428	0.031***
Used a coyote	0.677	0.468	0.641	0.480	0.737	0.440	0.096***
People traveling together	5.575	9.303	6.695	9.965	3.674	7.692	−3.021***
Traveled with children	0.094	0.291	0.111	0.315	0.063	0.244	−0.048***
Previous number of crossings	0.467	0.877	0.501	0.907	0.408	0.819	−0.092***
Country of origin							
El Salvador	0.284	0.451	0.224	0.417	0.385	0.487	–
Guatemala	0.351	0.477	0.389	0.487	0.286	0.452	–
Honduras	0.366	0.482	0.387	0.487	0.329	0.470	–
Observations	18,390		11,151		7,239		

*Notes: – ^{a} Calculated using predicted travel costs plus the reported smuggler fees. – Pre-program defined as the interval between 1/1/2012 to 30/6/2014. Post-program defined as the interval between 1/7/2014 and 31/12/2016. – *** $p < 0.001$; ** $p < 0.01$; * $p < 0.05$; † $p < 0.1$.*

Table A4: Effect of Southern Border Plan on the Intent to Remigrate by Sub-Sample

	I	II
**A. Gender**		
	Male	Female
SBP effect*Gender	-0.160^{***}	-0.165^{**}
	(0.034)	(0.057)
	[0.928]	
**B. Migration experience**		
	First	Multiple
SBP effect*Experience	-0.141^{**}	-0.177^{***}
	(0.045)	(0.038)
	[0.416]	
**C. Employment status**		
	Employed	Unemployed
SBP effect*Employed	-0.184^{***}	-0.140^{**}
	(0.038)	(0.042)
	[0.296]	
**D. Family in the US**		
	Yes	No
SBP effect*Network	-0.124^{**}	-0.229^{***}
	(0.040)	(0.036)
	[0.031]	
Observations	32,041	32,041

Notes: – Results are obtained from OLS regressions. – The regressions include the full set of control variables as in column VI of Table **??**, *and the interaction of the respective group indicators with the (i) treatment effect, (ii) time FE, and (iii) origin department FE. – Standard errors in parentheses (clustered at the origin-department level). The figures in squared brackets corresponds to the p-value for the test that the estimated coefficient is the same as in column I. –* *** $p < 0.001$; ** $p < 0.01$; * $p < 0.05$; † $p < 0.1$.
